# Supplementary material for: Caregiver Burden and Quality of Life in Late Stage Parkinson’s Disease
Source: Brain Sci. 2022 Jan 14;12(1):111. doi: 10.3390/brainsci12010111 (PMC8773513; doi:10.3390/brainsci12010111)
Supplement: Supplementary file 1 [file brainsci-12-00111-s001.zip › brainsci-1530214/brainsci-1530214.docx]

Principal investigators of the CLaSP Consortium:

Richard Dodel, University Hospital Essen, department of Geriatric Medicine, Germany; Stefan Lorenzl, Universität München-Klinikum Großhadern*,* Munich, Germany and Institute of Nursing Science and Practice, Salzburg, Austria; Wassilios G Meissner, CHU de Bordeaux, Service de Neurologie, Bordeaux, France, Université de Bordeaux, Institut des Maladies Neurodégénératives, Bordeaux, France and University of Otago, Department of Medicine, Christchurch, New Zealand and New Zealand Brain Research Institute, Christchurch, New Zealand; Bastiaan R Bloem, Radboud university medical centre; Donders Institute for Brain, Cognition and Behaviour and department of Neurology; Centre of Expertise for Parkinson & Movement Disorders, Nijmegen, the Netherlands; Joaquim J Ferreira, Universidade de Lisboa, Instituto de Medicina Molecular João Lobo Antunes, Faculdade de Medicina, Portugal.
